# How Did Conventional Nanoparticle-Mediated Photothermal Therapy Become “Hot” in Combination with Cancer Immunotherapy?

**DOI:** 10.3390/cancers14082044

**Published:** 2022-04-18

**Authors:** Wan Su Yun, Ji-Ho Park, Dong-Kwon Lim, Cheol-Hee Ahn, In-Cheol Sun, Kwangmeyung Kim

**Affiliations:** 1 KU-KIST Graduate School of Converging Science and Technology, Korea University, 145 Anam-ro, Seoul 02841, Korea; ip9801@kist.re.kr (W.S.Y.); dklim@korea.ac.kr (D.-K.L.); 2 NanoBio Materials Laboratory, Department of Materials Science and Engineering, College of Engineering, Seoul National University, 1 Gwanak-ro, Seoul 08826, Korea; jikl1028@snu.ac.kr (J.-H.P.); chahn@snu.ac.kr (C.-H.A.); 3 Medicinal Materials Research Center, Biomedical Research Division, Korea Institute of Science and Technology, 5, Seoul 02792, Korea

**Keywords:** photothermal therapy, immunotherapy, cancer

## Abstract

**Simple Summary:**

Photothermal therapy (PTT) has become effective through the development of nanoparticle-based photoabsorbers with various functions, such as targeting properties, high light-to-heat conversion, and photostability. Conventional nanoparticle-mediated PTT has attained localized efficiency in cancer treatment by heat-induced apoptosis or necrosis of cancer cells. Currently, such treatment methods evolve into cancer immunotherapy through the induction of immunogenic cell death (ICD). Damage-associated molecular patterns from dead cells by nanoparticle-mediated PTT activate immune cells for systemic anti-cancer effect. In this review, we investigate various nanoparticle-based PTT and compare its methodology to clarify how it undergoes a transition from thermotherapy to immunotherapy.

**Abstract:**

One of the promising cancer treatment methods is photothermal therapy (PTT), which has achieved good therapeutic efficiency through nanoparticle-based photoabsorbers. Because of the various functions of nanoparticles, such as targeting properties, high light-to-heat conversion, and photostability, nanoparticle-mediated PTT successfully induces photothermal damage in tumor tissues with minimal side effects on surrounding healthy tissues. The therapeutic efficacy of PTT originates from cell membrane disruption, protein denaturation, and DNA damage by light-induced heat, but these biological impacts only influence localized tumor areas. This conventional nanoparticle-mediated PTT still attracts attention as a novel cancer immunotherapy, because PTT causes immune responses against cancer. PTT-induced immunogenic cell death activates immune cells for systemic anti-cancer effect. Additionally, the excellent compatibility of PTT with other treatment methods (e.g., chemotherapy and immune checkpoint blockade therapy) reinforces the therapeutic efficacy of PTT as combined immunotherapy. In this review, we investigate various PTT agents of nanoparticles and compare their applications to reveal how nanoparticle-mediated PTT undergoes a transition from thermotherapy to immunotherapy.

## 1. Introduction

Since ancient Egyptians treated breast cancer with a fire drill [1], thermal therapy has been one of the important therapeutic methods against cancer. Thermal therapy has continuously broadened its applications in cancer treatment using a variety of energy sources for heating, such as radiofrequency [2,3], microwaves [4,5], focused ultrasound [6,7], and laser light [8,9]. In particular, the development of nanoparticles combined with a laser enables a breakthrough that overcomes the limitations of traditional thermal therapy. Monochromatic and coherent laser beams deliver high energy into a narrow target area with good precision and minimal power loss [10]. In response to laser irradiation, tumor-targeting nanoparticles with a photothermal effect increase anti-cancer therapeutic efficacy and reduce collateral damage to the surrounding healthy tissues [11]. Today, researchers have realized the versatility of nanoparticle-mediated photothermal therapy (PTT) in cancer immunotherapy, using anti-cancer immune reactions induced by PTT [12].

### 1.1. Nanoparticle-Mediated PTT

Traditional PTT used to be an ineffective therapeutic method except for in superficial tumors because of its non-selectivity and low penetration depth. The penetration depth of visible light for treatment is not deep enough to reach cancers in deep tissues [13]. Moreover, light-induced heat causes unwanted damage in normal tissues due to the non-specific absorption by healthy tissues [14]. However, the emergence of nanoparticle-based therapeutic agents for PTT significantly strengthens their therapeutic efficacy of PTT. The enhanced biostability of nanoparticles allows them to circulate for a prolonged time until they arrive at tumor sites. Because of the incomplete structure of the blood vessels of tumors, the nanoparticles extravasate and accumulate in the tumor, which is called the enhanced permeability and retention (EPR) effect [15]. In contrast to this passive targeting, the active targeting strategy achieves selective accumulation of nanoparticles through surface modification of various targeting moieties that interact with overexpressed biomarkers of tumors. The sophisticated design of nanoparticles with targeting or self-assembling properties at tumors expedites the accumulation in tumor tissues [16,17]. The selective accumulation of nanoparticle agents is critical for the confined therapeutic effect of PTT in the tumor area and the minimized damage to surrounding healthy tissues. Furthermore, nanoparticles with tunable optical properties are able to absorb near-infrared (NIR) laser and generate heat in deep tissues. This NIR range is called the first biological window, and extends from 700 nm to 1000 nm between the absorption band of hemoglobin (540 nm and 576 nm) and water (980 nm) [14]. Because of the low extinction of NIR light in the body, nanoparticle agents for PTT absorb light energy in deeper locations and efficiently convert it into heat. Therefore, this passive or active targeting of nanoparticle-based PTT agents with NIR absorption overcomes the poor reliability of traditional PTT.

After the accumulation of nanoparticle-based agents for PTT in the tumor, laser irradiation on the nanoparticle agents initiates anti-cancer effects differently depending on the operating mode of the laser. A pulsed wave (PW) laser periodically delivers high intensity to the PTT agents and produces heat around them. This localized heat evaporates a thin layer of water and creates bubbles, expanding and collapsing near PTT agents [18]. The origin of cellular damage is the mechanical stress by cavitating bubble formation [19], not heat by PW laser irradiation because heat diffusion is restricted to the vicinity of PTT agents [14,20]. In contrast, heat is the most dominant cause of cell death in the case of the irradiation with continuous wave (CW) lasers. When a CW laser excites electrons, they transfer light energy to the surrounding medium through the relaxation of nonradiative decay [21]. This energy conversion increases the temperature and instigates a hostile environment in tumor tissues.

The characteristics of photothermal damage on tumors are dependent on the temperature. Above 55 °C, various hazardous changes, such as protein denature, edema, and mitochondrial swelling, occur within a few minutes of treatment [22]. In particular, proteins denature instantly above 60 °C, and water vaporization induces tissue explosion above 80 °C [14]. However, such drastic changes also cause cell death in healthy tissues because of uncontrollable heat diffusion from therapy sites to the surrounding area. Moreover, this radical photothermal treatment leads directly to irreversible cell death with inflammation, called necrosis [23,24]. After necrotic death, cells release various inflammatory cytokines and activate the immune system, resulting in inflammation. Necrosis is undesirable because the inflammation is thought to exacerbate the therapeutic efficacy of PTT and inhibit tumor regression [25]. Researchers also regard metastasis and recurrence of cancer to be due to the death of the surrounding tissues [26]. Therefore, various work on nanoparticle-based PTT has suggested therapeutic approaches with lower temperatures [27].

The threshold of PTT with low temperature is generally below 48 °C, at which point hyperthermia occurs. Even though hyperthermia also induces cell death by protein denature, cell membrane disintegration, and the impairment of DNA/RNA synthesis, it requires a longer duration of treatment, compared with the irreversible photothermal damage at high temperatures [28]. For example, the cytotoxic effect appears within 3~4 min if the treatment temperature is above 70 °C, while hyperthermia takes effect after an hour at 42 °C [29]. The hyperthermic environment affects various processes at the cellular level according to the magnitude of temperature increment. The onset temperature of protein denature is 40 °C [30], and the synthesis of heat shock protein begins to be inhibited above 41 °C [31]. If heat shock proteins fail to respond to the aggregation of denatured proteins, cells become susceptible to cell death because of the inactivation of the DNA repair process, and cell cycle progression [32]. If the temperature increases up to 43~45 °C, oxidative stress on the cell membrane exacerbates the cell structure [33,34]. Then, both apoptosis and necrosis happen in the range of 45~48 °C [35].

Conventionally, the control of treatment temperature is important in PTT because it may induce apoptotic cell death without inflammation during the treatment. The various nanoparticle-based PTT demonstrate the ability to adjust the treatment temperature by controlling the properties of nanoparticles (e.g., size, concentration, and optical absorption) and energy sources (e.g., laser intensity, wavelength, and irradiation time). Therefore, abundant studies on nanoparticle-based PTT with low energy radiation focus on the fine-tuning of the treatment condition for facilitating apoptosis, instead of necrosis, to avoid inflammatory responses [36]. In addition, such active studies are leading to the development of numerous nanoparticle-based PTT agents with low toxicity, biocompatibility, selective tumor targeting, large optical absorption, and efficient light conversion into heat for successful biomedical applications. However, this localized treatment may fail to treat metastasis or regional primary tumors that are out of the irradiated area [37]. Therefore, systemic anti-cancer effects of PTT gain attention as promising therapeutic methods.

### 1.2. Cancer Immunotherapy with Nanoparticle-Mediated PTT

As mentioned previously, the therapeutic efficacy of PTT originates from hyperthermia, which induces protein denaturation, DNA damage, and disruption of cell membrane integrity [38]. These biological impacts can stimulate the release of damage-associated molecular patterns (DAMPs) from treated cancer cells. Instead of longing for apoptosis without immune reactions, cancer immunotherapy with nanoparticle-mediated PTT utilizes such DAMPs for cancer treatment. PTT induces immunogenic cell death (ICD), a form of regulated cell death that causes systemic anti-cancer immune reactions [38,39,40,41]. If cancer cells experience stress, injury, or death after PTT, they release DAMPs, such as heat shock proteins (Hsp), adenosine triphosphate (ATP), high-mobility group box 1 (HMGB1), and calreticulin (CRT). These DAMPs inside the cancer cells exist in a non-immunological state until they are released from the cells [42]. After the release of DAMPs (e.g., ATP and HMGB1) or translocation across the plasma membrane (e.g., CRT and Hsp90), these biomarkers play a role as danger signals or adjuvants that activate the innate immune system and recruit various immune cells [43,44]. Specifically, dendritic cells (DCs) engulf cancer cells with DAMPs and activate cytotoxic T-cells (CTLs), which systemically kill cancer cells with the same type of DAMPs [45,46,47]. Therefore, ICD by PTT works as in situ vaccination against cancer and provides systemic anti-cancer effects.

Various nanoparticle-based photoabsorbers allow us to achieve ICD-inducing PTT. For example, melanin [48], Prussian blue [41], iron oxide [23], and gold nanoparticles [49] have demonstrated their excellent potential as ICD inducers for cancer immunotherapy. Their photothermal effect has successfully removed primary tumors, as well as metastatic tumors through systemic immune responses of DAMP-specific lymphocytes against cancer. The tumor-targeting properties of these nanoparticles is also necessary for tumor-specific immune responses. Nanoparticles selectively accumulate in tumors through passive targeting as a result of the extravasation of nanoparticles through the leaky vasculature of tumors [50]. In contrast, the active targeting strategy utilizes a surface modification of nanoparticles with various targeting moieties, such as peptides, antibodies, carbohydrates, to interact with overexpressed biomarkers in tumors [51]. This tumor-targeting properties leads to tumor-specific ICD by nanoparticle-based PTT for effective anti-cancer immune responses. Furthermore, excellent light conversion ability into heat, stability, and biocompatibility are essential requirements for the successful applications of PTT in cancer immunotherapy.

**Figure 1:**
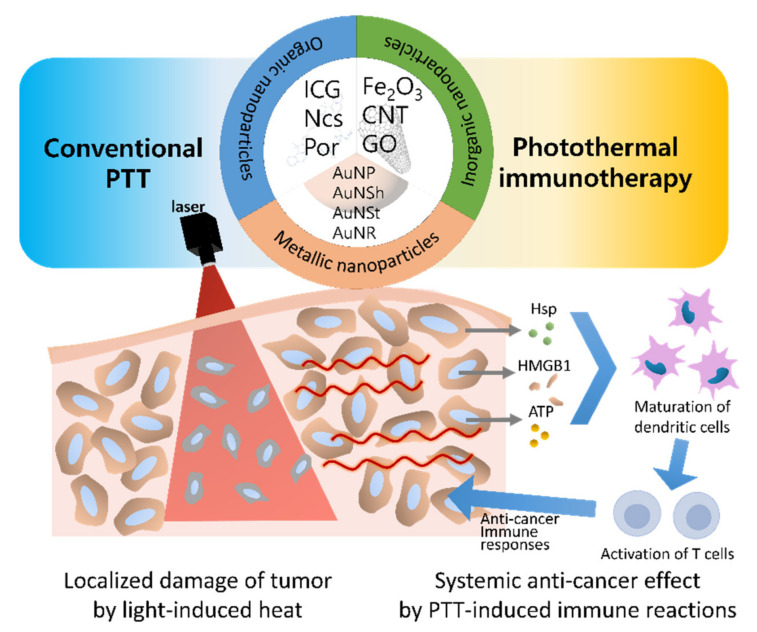
Schematic diagram of nanoparticles for photothermal therapy (PTT) according to the therapeutic mechanisms. Conventional PTT gives priority to the cell death (necrosis or apoptosis) of targeted cancer in the radiated area. In contrast, photothermal immunotherapy focuses on the immune reactions after PTT for systemic anti-cancer effect. (Abbreviations: PTT—photothermal therapy; ICG—indocyanine green; Ncs—naphthalocyanines; Por—porphyrin; CNT—carbon nanotubes; GO—graphene oxide; AuNP—gold nanoparticles; AuNSh—gold nanoshells; AuNSt—gold nanostars; AuNR—gold nanorods; Hsp—heat shock protein; HMGB1—high-mobility group box 1; ATP—adenosine triphosphate).

These properties of nanoparticles are desirable for overcoming the limitations of conventional ICD inducers, such as anti-cancer drugs (e.g., oxaliplatin, cyclophosphamide, and anthracyclines) and cardiac glycosides (e.g., digoxin, digitoxin, ouabain, and lanatoside C) because they have a poor circulation time, low bioavailability, non-specific accumulation, and undesirable toxicity [52,53,54,55]. Therefore, the development of nanoparticle-based PTT agents and their applications in cancer immunotherapy become an active research area. In this review, we introduce nanoparticle-mediated PTT that overcome the limitations of conventional PTT, and their evolution to cancer immunotherapy (Figure 1).

## 2. Photothermal Therapy with Organic-Dye Nanoparticles

For effective treatment against cancer, exogenous PTT agents with NIR absorption are indispensable for the deeper penetration depth and efficient heat generation. Several NIR organic dyes, such as indocyanine green (ICG), naphthalocyanine, and porphyrin, have desirable optical properties for biological applications in PTT [56,57,58,59,60,61,62,63,64]. In addition, they emit fluorescent signals in the NIR range, which provide biological information in the body with minimal obstruction. These properties have made organic dyes an attractive choice as PTT agents. However, their drawbacks often impede their transition into practical bioapplications [65]. First of all, the photostability of organic dyes is not robust enough to sustainably produce heat under light exposure. Even worse, the light-induced heat from organic dyes exacerbates the stability of their chemical structure. Not only light and heat, but also the aqueous environment and biological conditions in the body amplify the degradation of organic dyes and reduce the bioavailability of organic dyes in PTT.

**Figure 2:**
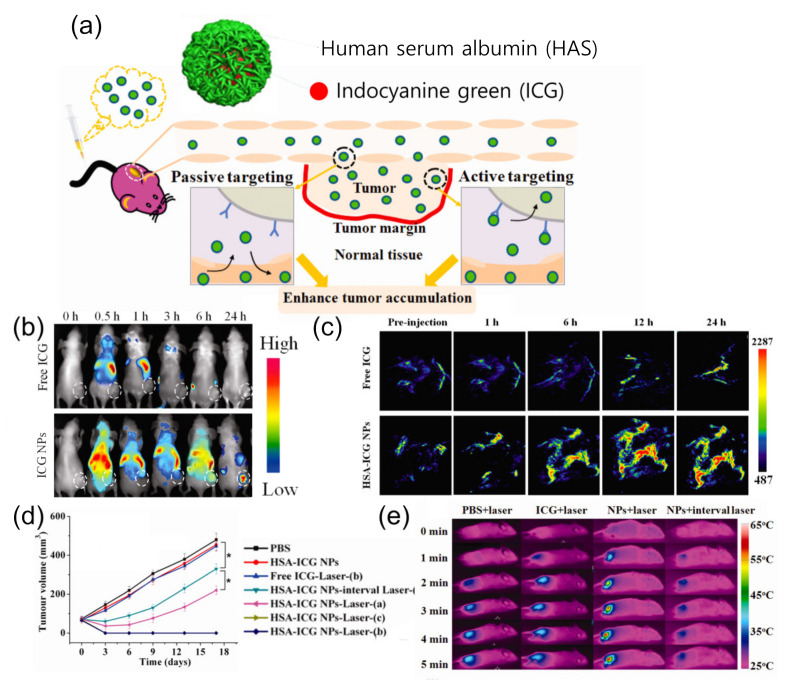
(**a**) Scheme of HSA-ICG nanoparticles for enhanced tumor accumulation in 4T1 tumors by passive (EPR effect) and active (gp60 transcytosis pathway) targeting; (**b**) fluorescence imaging of 4T1 tumor-bearing mice after the injection of free ICG (upper) and HSA-ICG nanoparticles (lower) at different time intervals; (**c**) photoacoustic imaging of the mice; (**d**) tumor growth curves after different treatments (* *p* < 0.05, a: partial irradiation, b: accurate irradiation, c: over irradiation); (**e**) thermal images of mice exposed to 808 nm laser for 5 min. Figures are reproduced from ref. [58] with permission from the American Chemical Society.

Nanoparticles containing such organic dyes enhance the stability and bioavailability of organic dyes for effective PTT [66]. Nanoparticle carriers protect loaded organic dyes from light, heat, and biomolecules to maintain their chemical structure. Additionally, the long circulation time and tumor-targeting properties improve the bioavailability until they reach the tumor tissues and exhibit their therapeutic effect upon laser irradiation. The enhanced stability and bioavailability of ICG was demonstrated through the encapsulation with lipid polymer nanoparticles [56,57]. The poor aqueous stability and rapid elimination of free ICG were successfully overcome by the nanoparticle formulation. These ICG-loaded nanoparticles had a photothermal effect that increased intratumoral temperature to 50 °C and induced necrosis upon 808 nm laser irradiation. In particular, ICG-loaded human serum albumin (HSA-ICG) nanoparticles showed enhanced cellular uptake and tumor-targeting properties due to the expressed albumin receptors of cancer cells (Figure 2a) [58]. The selective accumulation of HSA-ICG nanoparticles was conspicuous when the biodistribution was compared with that of free ICG (Figure 2b). The tumor-targeting properties were advantageous not only for therapy but also for imaging. Because of fluorescence and photoacoustic property, HSA-ICG nanoparticles visualize tumor tissue with both fluorescent and photoacoustic imaging techniques (Figure 2c). In addition, the flow cytometry of 4T1 cells labeled with propidium iodide and Annexin V-Alexa Fluor 488 proved late apoptosis/necrosis of cancer cells after PTT. As a result, tumor growth was significantly inhibited due to necrosis of tumor tissue induced by the increased temperature at 57 °C upon 808 nm laser irradiation (Figure 2d,e).

Likewise, the therapeutic outcome of naphthalocyanine and porphyrin also improved in PTT if these organic dyes were applied in the form of nanoparticles. For example, naphthalocyanine formed nanoparticles with PEG-*block*-poly(Ɛ-caprolactone) [59] or Pluronic^®^ F-127 [60] polymers. These organic-dye nanoparticles showed excellent inhibition of tumor growth upon NIR laser irradiation. Additionally, these nanoparticles were also capable of fluorescence or photoacoustic imaging for theragnostic applications. Lam et al. reported a porphyrin nanoparticle platform for various imaging and therapeutic functions [61]. The porphyrin nanoparticles increased in temperature to 57 °C under 690 nm laser irradiation and induced necrosis of tumors.

Organic-dye nanoparticles for PTT enabled easy transition to photothermal immunotherapy. The most important treatment condition for the applications of PTT in immunotherapy is temperature. Because the aim of conventional PTT was centered on cell death, the treatment temperature could be either over 50 °C for necrosis [56,57,58] or below 50 °C for apoptosis of cancer cells [64,67]. In contrast, photothermal immunotherapy requires scrupulous checking and control of temperature to maximize the photothermal damage to tumor tissues and produce DAMPs for systemic anti-cancer immune reactions [68]. Simultaneously, the light-induced heat should minimize the adverse effects on surrounding tissues and the immune system for effective therapeutic effects. Moreover, histological analysis of photothermal immunotherapy focuses on not only the localized cell death in the tumor, but also the various DAMPs and systemic activation of immune cells.

**Figure 3:**
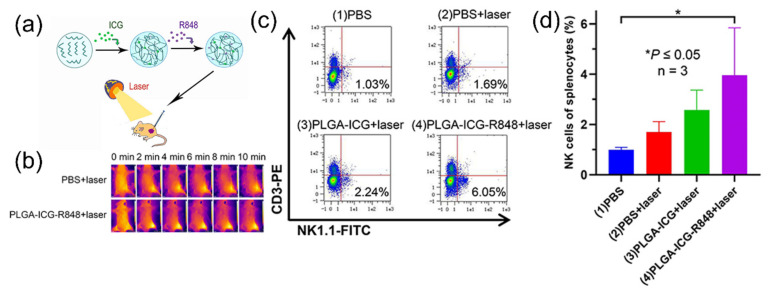
(**a**) Schematic diagram of PLGA-ICG nanoparticles with R848 for photothermal immunotherapy against RM9-tumor models; (**b**) thermal images of mice after the subcutaneous injection, followed by laser irradiation; (**c**) flow cytometry displaying the population of natural killer cells after PTT; (**d**) increased population of natural killer cells after treatment (* *p* < 0.05, *n* = 3). Figures are reproduced from ref. [62] with permission from Dove Press.

The application of ICG nanoparticles to photothermal immunotherapy was demonstrated by Lin et al., who fabricated ICG nanoparticles with poly(lactic-*co*-glycolic acid) (PLGA) [62]. PLGA-ICG nanoparticles also contained resiquimod (R848), a Toll-like receptor 7 and 8 agonist that promotes cytokine upregulation (Figure 3a). Upon 808 nm laser irradiation (0.8 W/cm^2^,10 min), PLGA-ICG nanoparticles increased the temperature of the tumor up to 50 °C (Figure 3b). The light-induced heat destructed tumor cells, from which the released DAMPs maturate dendritic cells (DCs). Consequently, the maturated DCs activate natural killer cells, which was proved by the increased population of CD3-NK1.1+ cells (Figure 3c), as well as CD11c+CD86+ and CD11c+CD80+ cells. (Figure 3d). Similarly, Li et al. showed that nanofibers containing ICG were effective for the activation of immune cells, such as CD11c, CD80, and CD86, for anti-cancer effect upon 808 nm laser irradiation and inhibited tumor growth [63]. Therefore, nanoparticle-mediated PTT was an effective strategy for inducing systemic therapeutic effects with anti-cancer immune reactions.

## 3. Photothermal Therapy with Inorganic Nanoparticles

Inorganic nanoparticles have attracted attention for the application of PTT because of their unique optical and physicochemical properties. For example, iron oxide nanoparticles have distinct magnetic properties, which can be applied to a contrast agent of magnetic resonance imaging (MRI) [69] and a delivery carrier of anti-cancer drugs [70]. Moreover, iron oxide nanoparticles have been used as thermal therapy agents because they convert electromagnetic energy into heat in the alternating magnetic field [71]. Even though iron oxide nanoparticles also have a photothermal effect [72,73], their applications in PTT have been limited, because their absorption in the NIR range is not sufficient. However, such limitations would not hamper the application of iron oxide nanoparticles in PTT, because of their easy surface modification. Their application in thermal therapy can be extended to photothermal therapy because of their easy surface modification.

**Figure 4:**
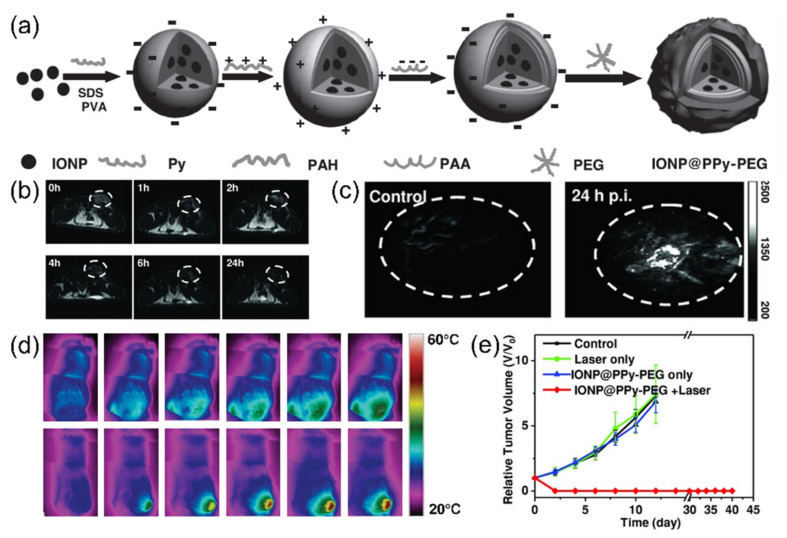
(**a**) Schematic illustration of the synthesis of IONP@PPy-PEG nanoparticles for 4T1 cancer treatment; (**b**) T2-weighted MR images of 4T1-bearing mice after intravenous injection of IONP@PPy-PEG (white circle: tumor sites); (**c**) photoacoustic images of tumor-bearing mice with IONP@PPy-PEG; (**d**) thermal images of mice with IONP@PPy-PEG after 808 nm laser irradiation; (**e**) growth curve of 4T1 tumors after treatment. Figures are reproduced from ref. [74] with permission from Wiley.

Song et al. developed PEGylated iron oxide nanoparticles doped with polypyrrole, an NIR-absorbing conjugated polymer (IONP@PPy-PEG), as a PTT agent (Figure 4a) [74]. IONP@PPy-PEG was prepared through a layer-by-layer method to obtain water solubility and stability. This excellent biocompatibility enabled the accumulation in the tumor after intravenous injection, which was proved by both MRI (Figure 4b) and photoacoustic imaging (Figure 4c). After 808 nm laser irradiation, the intratumoral temperature increased to 58 °C (Figure 4d), which left a burn on the irradiated tumor area. As a result, tumor volume was successfully suppressed after the treatment (Figure 4e).

Cylindrical graphene sheets, called carbon nanotubes (CNTs), can be used as PTT agents because of their strong NIR absorption. In particular, CNTs are appropriate nanomaterials as PTT agents because the absorption range and photothermal conversion of CNT are superior to that of gold or magnetic nanoparticles [75]. Additionally, PTT and chemotherapy have often been combined through carbon nanotubes, since their high surface area is suitable for drug delivery. Dong et al. functionalized multi-walled CNTs with chitosan to conjugate doxorubicin [76]. Upon NIR laser irradiation, CNTs increased the temperature of the tumor region to 67 °C. This therapeutic effect was accelerated by the release of doxorubicin in response to pH conditions. Single-walled CNTs may not be as efficient for drug delivery as multi-walled CNTs, because they have smaller dimensions and weaker interactions than multi-walled CNTs [77,78]. However, they also have strong NIR absorption properties for PTT. Therefore, single-walled CNTs were coated with various biocompatible polymers, such as PEG [79] and hyaluronic acid [80], and used as PTT agents. Upon laser irradiation, single-walled CNTs generated heat over 55 °C and caused photothermal ablation. As a result, tumor volume was significantly reduced by heat-induced necrosis. The use of single-walled CNTs would be advantageous for clinical applications because they can be cleared from the body within three months [78].

Graphene oxide also has excellent drug loading efficacy and photothermal effect. Its loading capacity can be as high as 200% and its photothermal properties are superior to those of CNTs [81]. Moreover, the broad absorption of graphene oxide in the NIR range, similar to carbon nanotubes, efficiently converts light energy into heat upon 808 nm laser irradiation. For example, Yang et al. synthesized PEGlylated nanographene sheets [82]. Their graphene oxide nanoparticles showed an excellent photothermal effect that caused ablation of tumors upon laser irradiation. Moreover, Zhang et al. combined chemotherapy and PTT, using the high drug loading efficiency of graphene oxide [81]. They conjugated doxorubicin on PEGlyated nanographene oxide nanoparticles, which displayed an absorption peak of doxorubicin at 490 nm. The toxicity of doxorubicin and the photothermal effect of graphene oxide nanoparticles synergistically enhanced the efficacy of cancer treatment.

As we have seen in organic dye nanoparticles, current applications of inorganic nanoparticles for PTT are also being directed towards immunotherapy. Previously, PTT with inorganic nanoparticles (e.g., iron oxide nanoparticles, CNTs, and graphene oxide) induced thermal ablation at temperatures above 50 °C [74,76,79,80,81,82]. Although the high heating temperature achieved a reduction of tumor volume, these treatment methods confined their therapeutic effect to within the irradiated area. This localized treatment had the risk of unwanted damage to healthy tissues and relapse of tumor due to the insufficiently wide irradiated area. In contrast, recent studies in which PTT has been performed with inorganic nanoparticles have concentrated on the systemic anti-cancer effect. For the regulated immune reactions, the treatment temperature was generally below 50 °C to avoid uncontrolled inflammation. In addition, histological analysis viewed systemic immunity against cancer.

**Figure 5:**
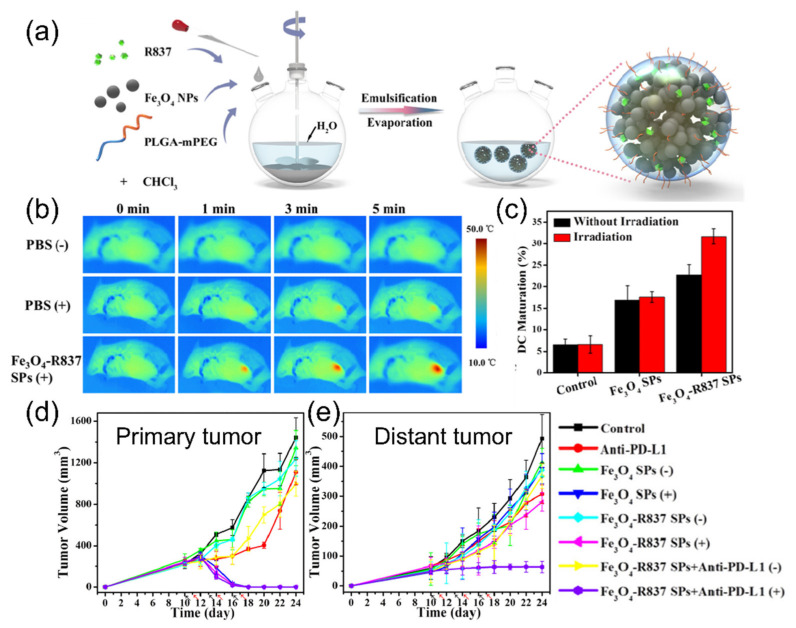
(**a**) Schematic illustration of iron oxide nanoparticles for immunotherapy against 4T1 models; (**b**) thermal images of 4T1 tumor-bearing mice after laser irradiation; (**c**) change of dendritic cell maturation after the treatment; (**d**,**e**) tumor growth curves of the primary (**d**) and metastatic tumors (**e**). Figures are reproduced from ref. [83] with permission from the American Chemical Society.

Ge et al. applied iron oxide nanoparticle immunotherapy [83]. Iron oxide nanoparticles were encapsulated in PLGA-PEG copolymer with imiquimod (R837), a Toll-like receptor 7 agonist (Figure 5a). Upon NIR laser (808 nm) irradiation, iron oxide nanoparticles destroyed the 4T1 breast tumor with a photothermal effect (Figure 5b) and triggered the release of tumor-associated antigens. This caused the maturation of dendritic cells for the activation of the immune system (Figure 5c). However, the photothermal effect of iron oxide alone was not effective enough to cause strong immune reactions due to the low extinction coefficient of iron oxide nanoparticles. Released by light-induced heat, R837 worked as an adjuvant and reinforced the effective anti-cancer immune responses. As a result, PTT and systemic anti-cancer immunity removed primary tumors and suppressed tumor metastasis (Figure 5d,e).

Nanoparticle-mediated photothermal immunotherapy with iron oxide nanoparticles often requires additional immunotherapeutic strategies because of their poor photothermal effect [83]. For example, Zhang et al. combined iron oxide nanoparticles with immune checkpoint blockade in a PLGA/PEG delivery platform [84]. They induced immunogenic cell death of cancer at mild temperature (45 °C) through the 660 nm laser irradiation of iron oxide nanoparticles. The optical droplet vaporization of the PTT agent after laser irradiation also caused the release of anti-programmed cell death-receptor 1 (PD1) antibodies, which prompt cancer cell death by tumor-specific T cells.

Moreover, CNTs and graphene oxide nanoparticles have often been combined with other immunotherapeutics. Wang et al. integrated multi-walled CNTs with synthetic oligodeoxynucleotides of CpG motifs, which promote various pro-inflammatory cytokines [85]. Together with the CpG adjuvant, the photothermal treatment at 46 °C with CNTs accomplished activation of CD4+ and CD8+ T cells. Similar to CNTs, it is possible for graphene oxide nanoparticles to accommodate indoleamine-2,3-dioxygenase (IDO) inhibitors [86]. IDO is an enzyme that catalyzes tryptophan to kynurenine. Because the lack of tryptophan leads to the inhibition of clonal expansion of T cells, tumors overexpress IDO to prevent anti-cancer immune reactions. Combining PTT, IDO inhibition, and PD-L1 immune checkpoint blockade with graphene oxide nanoparticles, Yan et al. enhanced tumor-infiltrating lymphocytes and reduced immune suppression by regulatory T cells. In short, the photothermal effect of inorganic nanoparticles makes them an attractive choice as therapeutic agents for photothermal immunotherapy. In addition, their excellent functionality enabled the combination with other immunotherapeutic methods for improved systemic anti-cancer immunity. Therefore, inorganic nanoparticles have shown their great potential as therapeutics for immunotherapy.

## 4. Photothermal Therapy with Metallic Nanoparticles

Gold nanoparticles have been a popular therapeutic agent for PTT because of their unique properties. The easy surface modification and controllable physicochemical properties of gold nanoparticles are valuable for various medical applications, not to mention their biocompatibility and low cytotoxicity [29,87,88,89,90,91,92]. In particular, PTT has benefited from the tunable absorption properties of gold nanoparticles. The absorption properties originate from the surface plasmon resonance, the collective oscillation of conduction electrons at the surface of gold nanoparticles. Because the particle size, shape, and composition affect the surface plasmon resonance, we can tune the optical absorption property to the wavelength of the excitation laser [93]. Consequently, the absorbed light energy is converted into heat through a series of non-radiative routes between electrons and phonons for the maximum photothermal output [94,95]. Therefore, various gold nanoparticles with diverse sizes and shapes have been applied to PTT.

Gold nanospheres have been investigated as a PTT agent because they efficiently generate heat upon laser irradiation with the wavelength of their surface plasmon resonance [96]. For example, 40 nm gold nanospheres were applied to the photothermal therapy of oral cancer cells in vitro [97]. However, the applications of gold nanospheres have been limited because their maximum absorption wavelength is at about 530 nm, which is outside the NIR range, and they possess a shallow penetration depth in vivo [98]. The absorption wavelength of gold nanospheres can be shifted into the NIR region through the aggregation or clustering of nanoparticles [99,100]. Using the aggregation-induced red-shift of surface plasmon resonance, Kang et al. adsorbed 30 nm spherical gold nanoparticles on graphene oxide sheets to shift the maximum absorption wavelength from 528 nm to 600 nm. After the injection of the sheet-attached nanoparticles into tumor-bearing mice, the temperature increased to 58 °C upon laser irradiation and necrosis of tumor tissue was observed.

Gold nanoshells and nanocages consist of a dielectric or hollow core with a thin wall of gold materials surrounding the core. Their tunable absorption properties originate from their particle size and the proportion of gold in the wall [101,102]. Therefore, gold nanoshells and nanocages with NIR absorption are also frequently used for PTT. Ke et al. designed 300 nm PEG-coated gold nanoshells as a PTT agent for the treatment of U87 human glioblastoma in nude mice [103]. Likewise, Chen et al. developed PEGlyated gold nanocages with the size of 48 nm for PTT [104]. After the irradiation of the 808 nm laser, they induced necrosis on the tumor at a temperature above 55 °C and successfully achieved a decrease in tumor growth by 70%.

Gold nanostars have a highly anisotropic shape, with NIR absorption as a result of the plasmon hybridization of the core and the tips [105]. The absorption wavelength can be precisely controlled by adjusting the size and thickness of the tip. Liu et al. synthesized 30 nm of gold nanostars that showed high accumulation and deep infiltration in tumors [106]. Then, the superior photothermal effect of gold nanostars induced thermal ablation after 4 min of 980 nm laser illumination at 50 °C. However, the light-induced heat can melt gold nanostars and transform them into nanospheres [107]. Therefore, surface modification of gold nanostars with silica was suggested for enhanced photostability [108].

**Figure 6:**
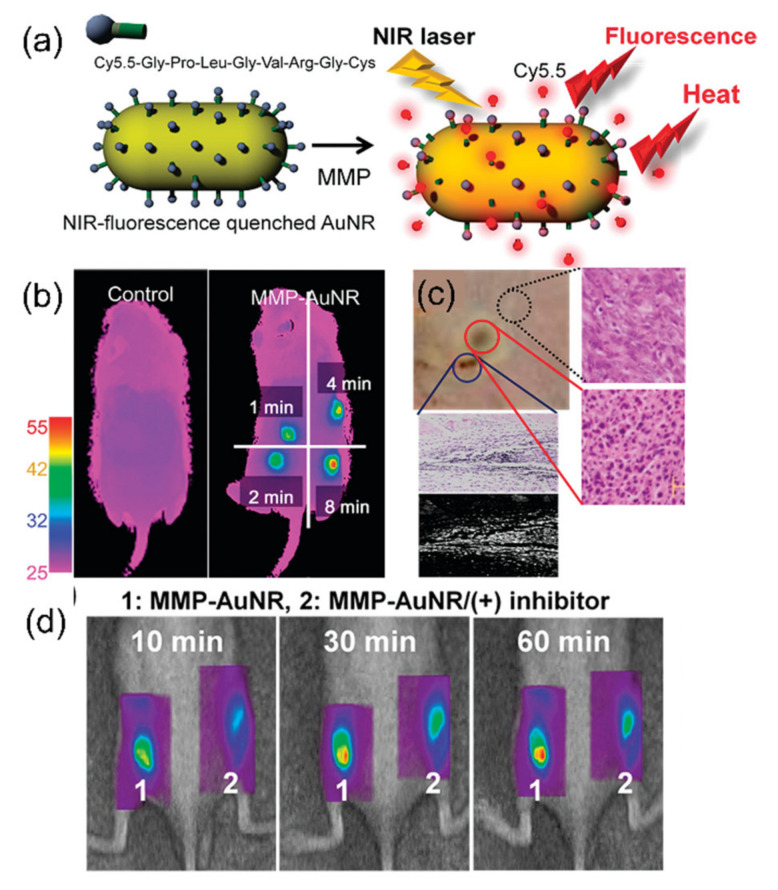
(**a**) Scheme of PTT agent with gold nanorods for SCC-7 tumor-specific imaging and therapy; (**b**) thermal images of tumor-bearing mice according to different laser irradiation times.; (**c**) histological analysis for tissue damage (right) and dark-field microscopic images of gold nanorods (below); (**d**) fluorescent tomographic images upon exposure to specific enzymes in cancer (1) and the inhibitor of the enzymes (2). Figures are reproduced from ref. [110] with permission from the American Chemical Society.

Compared with other gold nanoparticles, gold nanorods are peculiar because they exhibit two surface plasmon resonance peaks. Two different modes of plasmon oscillation (i.e., transverse and longitudinal directions) generate two absorption peaks, one at 530 nm and the other in the NIR region. The aspect ratio of gold nanorods is the critical factor that determines the absorption property [109]. Therefore, gold nanorods are synthesized with the proper aspect ratio to be in accordance with the wavelength of the excitation laser for PTT. Yi et al. created a multi-functional nanoparticle platform for simultaneous optical imaging and PTT (Figure 6a) [110]. They prepared gold nanorods with the maximum absorption at 670 nm, which coincided with the wavelength of the excitation laser. Upon laser irradiation, the temperature increased to 45 °C after 4 min (Figure 6b) and induced tissue damage (Figure 6c). In addition, this PTT agent also contained tumor-responsive optical probes that utilized the unique properties of gold nanorods. The fluorescence of organic dyes is quenched near gold nanoparticles due to the energy transfer from dyes to electrons on gold nanoparticles [91]. Then, the quenched fluorescence was restored in the tumor when the peptide linkers between gold nanorods and fluorescent dyes were degraded by an enzyme that overexpressed in the tumor (Figure 6d). Therefore, various unique properties of gold nanorods are suitable for the PTT agents with intriguing features.

In recent studies of PTT with gold nanoparticles, it has be destined to be accompanied by immunotherapy, as we have seen in the case of organic dye and inorganic nanoparticles. PTT with gold nanoparticles also promotes immune activation because of the death of tumor cells due to elevated heat release DAMPs and pro-inflammatory cytokines [49,111,112,113,114,115,116]. This ICD by PTT, occurring at a precisely controlled temperature below 50 °C, not at the conventional high temperature of 55 °C or more [41], can be easily achieved with gold nanoparticles, because optimization of their properties for the control of treatment temperature is relatively simple. Therefore, gold nanospheres [49], gold nanoshells [111], gold nanocages [112,113], and gold nanostars [114,115] have been applied in immunotherapy, bringing about systemic anti-cancer immune responses, along with the destruction of local primary tumors. These studies used NIR laser to increase the temperature to 50~52 °C, which lead to ICD for successful immunotherapy.

**Figure 7:**
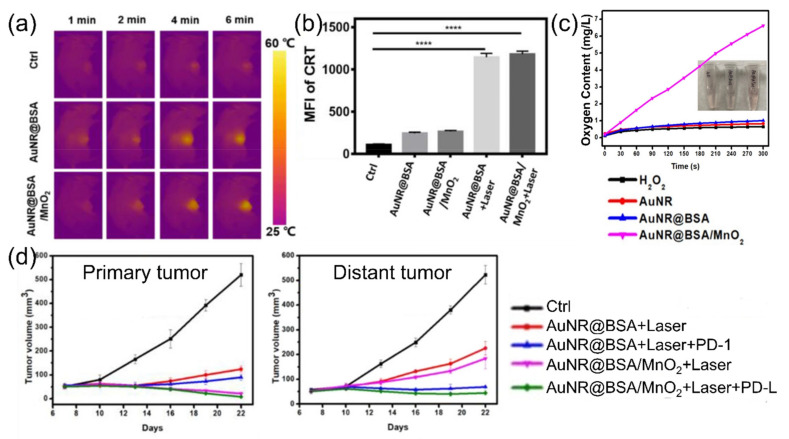
(**a**) Thermal images of 4T1 tumor area after laser irradiation; (**b**) expression of CRT after treatments (**** *p* < 0.0001); (**c**) oxygen generation curves from the decomposition of H_2_O_2_ in the presence of the PTT agent AuNRs; (**d**) tumor growth curve of primary and distant tumor after treatments (BSA: bovine serum albumin, PD-1: immune checkpoint blockade). Figures are reproduced from ref. [116] with permission from Wiley-VCH GmbH.

Previously, conventional PTT with gold nanorods aimed at thermal ablation of tumors [110]. In contrast, Zhou et al. designed multifunctional gold nanorods for PTT-induced ICD and cancer immunotherapy [116]. They modified the surface of gold nanorods with bovine serum albumin and MnO_2_ for biocompatibility and oxygen provision, respectively. Upon 808 nm laser irradiation, the temperature of the tumor area increased to 52 °C (Figure 7a), and induced overexpression of CRT, with one of the DAMPs indicating ICD (Figure 7b). After ICD by PTT, the MnO_2_ decomposed H_2_O_2_ into oxygen and relieved the hypoxic state of the tumor microenvironment (TME) (Figure 7c). As a result, the growth of both primary and distant tumors was significantly inhibited through the synergy of the photothermal effect of gold nanorods and the catalytic function of MnO_2_ (Figure 7d). To conclude, photothermal immunotherapy with gold nanoparticles enabled systemic anti-cancer effects that would overcome limitations of conventional PTT, including the relapse of tumors from the insufficiently irradiated areas and the restricted therapeutic effect.

## 5. Conclusions and Future Perspectives

Summaries of nanoparticle-mediated PTT and the advantages/disadvantages of nanoparticle agents are presented in Table 1 and Table 2, respectively. PTT has shown excellent performance in the treatment of localized cancer, with various advantages, such as minimal invasiveness, low cost, and high temporospatial control and selectivity [117]. The versatility of nanoparticles has reinforced conventional PTT through the development of nanoparticle-based PTT agents. The long circulation time and excellent bioavailability of nanoparticles increases the therapeutic effect while reducing unwanted damage to normal cells. Selective accumulation of PTT nano-agents with active/passive targeting properties in tumor tissue reduces the amount of drugs and undesirable side effects. Today, nanoparticle-based PTT is causing immunotherapy to adapt, shedding its skin of localized therapy. While conventional PTT pursued apoptosis, which does not induce immune reactions, current nanoparticle-based photothermal immunotherapy actively utilizes the released DAMPs in the hyperthermic environment for systemic anti-cancer immune reaction and alters TME for successful cancer treatment.

Furthermore, nanoparticle-based photothermal immunotherapy can easily be combined with other therapeutic methods [118,119]. A wide variety of nanoparticles with unique properties for PTT allow us to accommodate chemo-, radio-, immunotherapy, and even surgical treatment. Surgery is capable of removing solid tumors with large volumes, against which PTT may not be efficient because of its limited light penetration [120]. On the other hand, nanoparticle-mediated PTT could improve the clinical outcome of surgery by treating residual tumors, which may not be completely removed by surgeons. Therefore, PTT has developed as an adjuvant technique to surgery [120,121,122]. PTT nano-agents designed for controlled drug release, triggered by external NIR irradiation, would reduce the amount of drugs and unwanted exposure of drugs to normal cells [55]. In particular, the combination of PTT with chemo- or radiotherapy produces synergy, because PTT increases tumor vessel permeability, tumor perfusion, and oxygenation by hyperthermia and enhances the susceptibility of cancer to other treatment methods [123]. In addition, photodynamic therapy (PDT) could benefit from PTT because elevated temperature results in increased blood flow and improved hypoxia in TME for better PDT treatment [117]. Because both PTT and PDT can share the same light sources, and their different therapeutic mechanisms complement each other, nanoparticles are often applied to both PDT and PTT, simultaneously. Moreover, PTT is often combined with immune checkpoint blockade or immunoadjuvants on nanomaterials to supplement the insufficient amount of tumor antigen or DAMPs delivered by PTT alone. Therefore, nanoparticle-based PTT has expandability and compatibility with other therapies for enhanced therapeutic effects [55].

However, clinical applications of nanoparticle-based PTT are still limited, compared with the massive number of preclinical studies. Only a few particles, such as iron nanoparticles (for prostate cancer, ClinicalTrials.gov Identifier: NCT05010759) and gold nanoparticles (for head and neck cancer, ClinicalTrials.gov Identifier: NCT00848042; for prostate cancer, ClinicalTrials.gov Identifier: NCT04240639) are in clinical trials, even though numerous works have demonstrated that iron oxide or gold nanoparticles produce encouraging results in cancer treatment [25,124]. The penetration depth of light in the body is also an important factor that decides the therapeutic efficacy of nanoparticle-based PTT. The optimization of laser parameters, e.g., wavelength in the NIR range and high pulse frequency, could increase the penetration depth up to a range of several centimeters for the treatment of tumors in deep sites [125,126]. The complexity of the immune system and TME often frustrates our efforts to develop effective nanoparticle-mediated photothermal immunotherapy, which has the problems of large individual variations and low response rates [117,127,128]. Because of immunosuppressive TME, tumor heterogeneity, inappropriate functionalization of T cells, and deficient neoantigens of tumors, anti-cancer immune responses are often insufficient to treat tumors [129]. Most of all, there is no consensus on methodology in the application of nanoparticles. Nonetheless, nanoparticle-based PTT continuously proves its potential in cancer therapy, and eventually will become one of the most effective cancer therapies.

## Figures and Tables

**Table 1:** Summary of nanoparticles for PTT.

PTT Agent (Formulation or Modification)		Properties		Treatment Temp.	Laser Wavelength, Duration	Tumor Model	Therapeutic Effect	Ref.
		Size (nm)	Absorption Max. (nm)					
**Organic dye nanoparticles**	ICG (PLGA, lipid, doxorubicin)	90	815 nm	53 °C	808 nm, 5 min	MCF-7	Apoptosis	[56]
	ICG (cholesterol, lipid, folic acid)	20~40	810 nm	50 °C	808 nm, 5 min	MCF-7	Necrosis	[57]
	ICG (human serum albumin)	80	816 nm	57 °C	785 nm, 5 min	4T1	Necrosis	[58]
	Naphthalocyanine (PEG-PCL, Si)	40	785 nm	47 °C	785 nm, 10 min	A2780/AD	Apoptosis	[59]
	Naphthalocyanine (F127)	30	860 nm	60 °C	860 nm, 10 min	4T1	Photothermal ablation	[60]
	Porphyrin (Dendrimer form + PEG)	20	650–690	57 °C	690 nm, 2 min	SKOV3	Necrosis	[61]
	ICG (PLGA, R848)	160	780 nm	50 °C	808 nm, 10 min	RM9	Immune response	[62]
	ICG (thymopentin)	30	broad	47 °C	808 nm, 10 min	Pan02	Immune response	[63]
**Inorganic nanoparticles**	Iron oxide nanoparticle	20	broad	56 °C	808 nm, 3 min	A549	Apoptosis	[72]
	Iron oxide nanoparticle (doxorubicin)	10–310	480 nm (DOX)	57 °C	808 nm, 3 min	MCF-7 S180	Apoptosis and necrosis	[73]
	Iron oxide nanoparticle (polypyrrole)	100	broad	58 °C	808 nm, 5 min	4T1	Photothermal ablation	[74]
	Carbon nanotube, MW (chitosan, doxorubicin)	250	broad	67 °C	808 nm, 5 min	Bel-7402	Photothermal ablation	[76]
	Carbon nanotube, SW (PEG)	-	borad	55 °C	808 nm, 10 min	4T1	Photothermal ablation	[79]
	Carbon nanotube, SW (hyaluronic acid, cholanic acid, PEG, ICG)	390	broad	55 °C	808 nm, 10 min	SCC-7	Necrosis	[80]
	Graphene oxide (PEG)	10~50	broad	50 °C	808 nm, 5 min	4T1	Photothermal ablation	[82]
	Iron oxide nanoparticle (PLGA-PEG, R837,)	150	320 nm	<50 °C	808 nm, 10 min	4T1	Immune respones	[83]
	Iron oxide nanoparticle (PLGA-PEG, pentafluoropentane, anti-PD-1)	220	700 nm	45 °C	660 nm, 10 min	B16F10	ICD	[84]
	Carbon nanotube, MW (CpG or doxorubicin)	200	480 nm (DOX)	46 °C	808 nm, 3 min	B16	ICD	[85]
	Graphene oxide (IDO inhibitor, anti-PD-L1)	200	broad	53 °C	808 nm, 8 min	CT26	Immune response	[86]
**Metallic nanoparticles**	Gold nanosphere (anti-EGFR antibody)	40	530 nm	-	514 nm, 4 min	HSC 313 HSC 3	Photothermal ablation	[97]
	Gold nanoshell (PEG)	300	550 nm	60 °C	808 nm, 10 min	U-87 MG	Necrosis	[103]
	Gold nanocage (PEG)	90	800 nm	54 °C	808 nm, 10 min	U87MGwtEGFR	Necrosis	[104]
	Gold nanostar (PEG)	30	945 nm	50 °C	980 nm, 10 min	Sarcoma	Necrosis	[106]
	Gold nanorod (peptide, Cy5.5)	-	670 nm	45 °C	670 nm, 4 min	SCC-7	Photothermal ablation	[110]
	Gold nanosphere (liposome)	100	964 nm	50 °C	1064 nm, 10 min	4T1	ICD	[49]
	Gold nanoshell (PEG)	40	808 nm	-	808 nm, 3 min	B16-F10	ICD	[111]
	Gold nanocage (anti-PDL1, galunisertib)	60	800 nm	45 °C	808 nm, 10 min	CT26	ICD	[112]
	Gold nanocage (MnO_2_)	90	740 nm	50 °C	808 nm, 3 min	4T1	ICD	[113]
	Gold nanostar (selenium)	120	850 nm	52 °C	808 nm, 10 min	U14	ICD	[114]
	Gold nanostar (doxorubicin)	150	775 nm	<50 °C	808 nm, 5 min	CT26	ICD	[115]
	Gold nanorod (MnO_2_)	80 × 20	808 nm	50 °C	808 nm, 5 min	4T1	ICD	[116]

**Table 2:** Advantages and disadvantages of nanoparticle agents for PTT.

		Advantages	Disadvantages
Organic dye		- Simultaneous imaging during PTT - Excitation/Emission in NIR range - Clearance from body - Clinical applications (ICG)	- Low photostability - Lack of hydrophilicity - Low bioavailability
Inorganic Metallic	nanoparticles	- Excellent photostability - Easy synthesis with various sizes, and shapes - Simple surface modification - Enhanced tumor targeting - Tunable optical property (gold NP)	- Lack of biodegradability - Limited clinical applications - Absence of standardization in using nanoparticles
